# Treatment of Soil Contaminated by Mining Activities to Prevent Contamination by Encapsulation in Ceramic Construction Materials

**DOI:** 10.3390/ma14226740

**Published:** 2021-11-09

**Authors:** Juan María Terrones-Saeta, Jorge Suárez-Macías, Antonio Bernardo-Sánchez, Laura Álvarez de Prado, Marta Menéndez Fernández, Francisco Antonio Corpas-Iglesias

**Affiliations:** 1 Research Group TEP-222 “Materials and Mining Engineering”, Higher Polytechnic School of Linares, Scientific and Technological Campus of Linares, University of Jaén, 23700 Linares, Spain; jsuarez@ujaen.es (J.S.-M.); facorpas@ujaen.es (F.A.C.-I.); 2 Department of Mining, Topography and Structures, University of León, 24071 León, Spain; antonio.bernardo@unileon.es (A.B.-S.); laura.alvarez@unileon.es (L.Á.d.P.); marta.menendez@unileon.es (M.M.F.)

**Keywords:** retention of potentially toxic elements, potentially toxic elements, mining-contaminated soils, mining waste, ceramics, circular economy, sustainability, construction materials

## Abstract

Mining is an essential activity for obtaining materials necessary for the well-being and development of society. However, this activity produces important environmental impacts that must be controlled. More specifically, there are different soils near new or abandoned mining productions that have been contaminated with potentially toxic elements, and currently represent an important environmental problem. In this research, a contaminated soil from the mining district of Linares was studied for its use as a raw material for the conforming of ceramic materials, bricks, dedicated to construction. Firstly, the contaminated soil was chemically and physically characterized in order to evaluate its suitability. Subsequently, different families of samples were conformed with different percentages of clay and contaminated soil. Finally, the conformed ceramics were physically and mechanically characterized to examine the variation produced in the ceramic material by the incorporation of the contaminated soil. In addition, in this research, leachate tests were performed according to the TCLP method determining whether encapsulation of potentially toxic elements in the soil occurs. The results showed that all families of ceramic materials have acceptable physical properties, with a soil percentage of less than 80% being acceptable to obtain adequate mechanical properties and a maximum of 70% of contaminated soil to obtain acceptable leachate according to EPA regulations. Therefore, the maximum percentage of contaminated soil that can be incorporated into the ceramic material is 70% in order to comply with all standards. Consequently, this research not only avoids the contamination that contaminated soil can produce, but also valorizes this element as a raw material for new materials, avoiding the extraction of clay and reducing the environmental impact.

## 1. Introduction

Mining is one of the main industrial activities for the development of the welfare of the world’s population [1,2,3,4]. Through this activity, essential resources such as water, metals, aggregates and a wide range of other materials are extracted. It is therefore an essential activity that must continue to be developed [5]. However, mining activities have a significant impact on the environment [6]. This impact is mainly due to the modification of ground and surface water, the interaction with fauna and flora, the modification of the landscape and, in addition, the generation of potentially polluting waste [7,8,9,10].

New environmental regulations now rigidly regulate and control all environmental impacts arising from mining activities [11]. However, the generation of waste is inherent in this sector. Therefore, much research has been carried out in which waste from mining activities are reused as raw materials for new materials [12,13,14], developing a new circular economy [15]. However, there is one element of essential environmental importance that has been overlooked for decades—soil [16].

Soil is a living and dynamic system, which, as mentioned above, has been little respected in the past. It acts as a filter, accumulating and controlling the transport of toxic elements. These toxic elements can derive from different activities, however, mining can be one of the most damaging [17]. This is because soil contamination by toxic elements, mainly metals, is a serious environmental problem, as unlike other organic pollutants, these elements are not biodegrade and possess a long residence time in the soil [18,19,20]. 

Consequently, soil can be considered as contaminated by various factors or industrial activities. In particular, soil in the proximity of mining activities can be contaminated mainly by the leaching of mining waste [21]. These mining wastes produced at all stages of the mining activity (ore extraction, ore beneficiation and metallurgical extraction) are in most cases deposited in the open air. The contact of these wastes with atmospheric agents causes the leaching of toxic elements, resulting in the contamination of the soil, which acts as a filter.

The main toxic elements that can contaminate soil are arsenic, cadmium, chromium, copper, mercury, nickel, lead, tin, titanium and zinc. These elements are produced in large quantities in mining activities, especially in the metallurgical extraction phase, and can also be potentially polluting even at low concentrations [22,23].

However, in order to qualify a contaminated soil, it is essential to detail two fundamental aspects. Firstly, mobility, i.e., the ease with which these toxic elements can be transferred to other media [24,25]. Secondly, bioavailability, the capacity of physico-chemical access that an element has to enter the biological processes of micro-organisms [26]. Both aspects are controlled by the new regulations, since the formation of contaminated soil by mining activities can greatly harm flora, fauna and even people, being transferred to other media such as surface and groundwater. However, decades ago, all these environmental aspects essential for the environment and human health were not taken into account. For this reason, there is currently a diversity of contaminated soils belonging to mining activities that were closed down decades ago, as is the case of the present investigation. These contaminated soils represent a very important environmental and health problem, which is why appropriate operations must be carried out for their treatment [27].

The main operations currently carried out for the treatment of contaminated soils are decontamination and stabilization. Decontamination is basically the removal of soil for subsequent deposition in controlled landfills that prevent the mobility and bioavailability of toxic elements. Stabilization, in turn, consists of techniques that prevent the mobility and bioavailability of toxic elements, including vitrification [28,29]. This technique consists of subjecting contaminated soil to temperatures between 1600 °C and 2000 °C to vitrify the material [30] and, consequently, eliminate its leaching. This technique, as well as decontamination by soil removal, is very effective but very expensive, making it uneconomical in some cases. Therefore, in the present research a novel procedure was developed in which the two techniques are combined. On the one hand, the contaminated soil is removed to avoid contamination of the area; and on the other hand, this contaminated soil is used to manufacture ceramic material, bricks, thus retaining the potentially toxic elements in the ceramic matrix. In this way, landfill deposition of the contaminated soil is reduced, soil removal costs are reduced because it will be raw material for new materials, vitrification of the contaminating elements is produced and, furthermore, the extraction of new raw materials for ceramic conforming for bricks is avoided. 

The contaminated soil receptor material, brick ceramics, was selected for different reasons. On the one hand, the construction sector is one of the most polluting sectors in existence today, consuming enormous quantities of raw materials and causing significant environmental impact. On the other hand, the ceramic industry for the manufacture of bricks is located very close to the contaminated soils belonging to the mining district of Linares; therefore, its reuse would be economical as it would not involve huge transport costs. In addition, the ceramic matrix of the bricks largely retains the toxic elements of the contaminated soil [31,32]. At the same time, the use of contaminated surface soil avoids the costly task of clay extraction, reducing the cost of the final material. Finally, the landfill of contaminated soil is avoided, as well as the environmental impact that this could produce, developing a new circular and environmental economy.

For the detailed purpose, in the present research, ceramics for bricks were developed with contaminated soil from the mining district of Linares. Firstly, the clay and the contaminated soil were physically and chemically characterized to determine their compatibility, as well as the potentially harmful elements for the ceramic present in the contaminated soil. Subsequently, different families of ceramics with varying percentages of clay and contaminating soil were conformed, subsequently determining the physical and mechanical properties of these ceramics and evaluating the effect produced in the final material by the incorporation of the contaminated soil. Finally, and with the aim to corroborate that potentially toxic elements are retained in the ceramic matrix, the leaching of the different families of conformed ceramics was evaluated.

## 2. Materials and Methods

This section describes the materials used in this research, mainly contaminated soil and clay. At the same time, the tests that make up the methodology are shown in an orderly fashion. These tests show sufficient results to be able to confirm the objective of the research, to develop ceramics that retain the potentially toxic elements of the contaminating soil.

### 2.1. Materials

The materials used in the present investigation were contaminated soil and clay. Representative samples of both materials were collected according to the UNE-EN 932-1 standards [33]. The samples of the selected materials were dried at a temperature of 105 ± 2 °C. In this way, the existing moisture in the samples could be removed. However, the existence of moisture in the material does not harm the industrial process, it must simply be taken into account in order to add the correct percentage of water for ceramic conforming. In this case, moisture is eliminated in order to avoid useless variables that disturb the results of the research, as they do not provide additional information.

The main materials of the research are described below.

#### 2.1.1. Contaminated Soil

The contaminated soil is located in the mining district of Linares, Spain. More specifically in the immediate area of the abandoned foundry “La Cruz”. This old foundry was mainly dedicated to the metallurgical extraction of the existing lead and extracted from the Linares mines. As a result, there are still abundant wastes from the smelting concentration process in the abandoned facilities.

**Figure 1:**
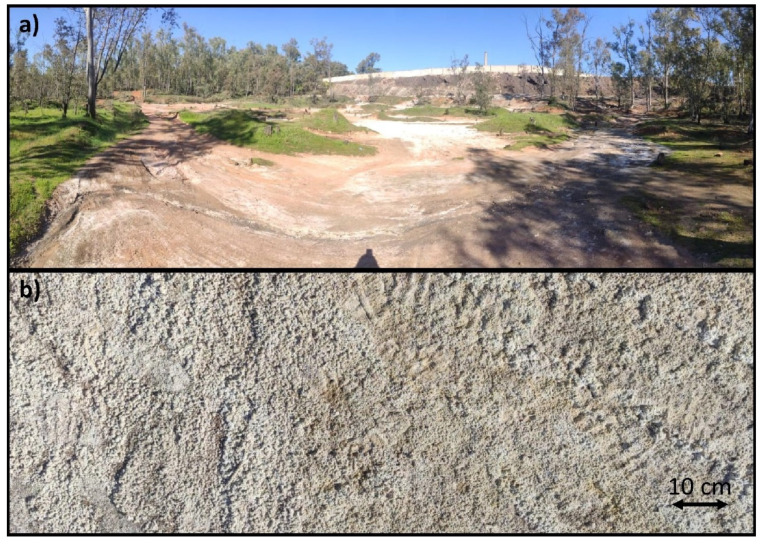
Contaminated soil in the Linares mining district: (**a**) general image of the area and (**b**) detailed image of the soil.

This waste seems to be the main cause of the contamination of the surrounding soil, as the location of this soil is at a lower level than the foundry installations. For this reason, it can be intuited that the leaching of the waste still existing in the abandoned foundry installations has caused the contamination of the lower soil level, as it has acted as a filter for the aforementioned leachate. The area of contaminated soil is approximately 30 square hectometers, so the environmental problem it currently poses is significant. Figure 1 shows the area, as well as a detailed image of the soil itself.

The soil samples were taken according to the UNE-EN 932-1 standards [33]. This sample was used directly in ceramic conforming after moisture removal, i.e., no grinding process was carried out. Only for chemical characterization, due to the requirements of the equipment used, the contaminated soil sample was dried and ground to a particle size of less than 100 micrometers.

#### 2.1.2. Clay

The clay used in this research for ceramic conforming with the contaminated soil was a red clay used in the manufacture of bricks. This type of clay is extracted and used by companies close to the mining district of Linares, Spain, where there is an important commercialization of ceramic elements, mainly bricks for construction. 

The clay sample was taken, as well as the contaminated soil, according to the UNE-EN 932-1 standards [33]. It was then dried and sieved through a 0.25 mm sieve, obtaining a material similar to that usually used in companies that use this material for the manufacture of bricks. The sample thus prepared was used for chemical characterization and ceramic conforming.

### 2.2. Methodology

**Figure 2:**
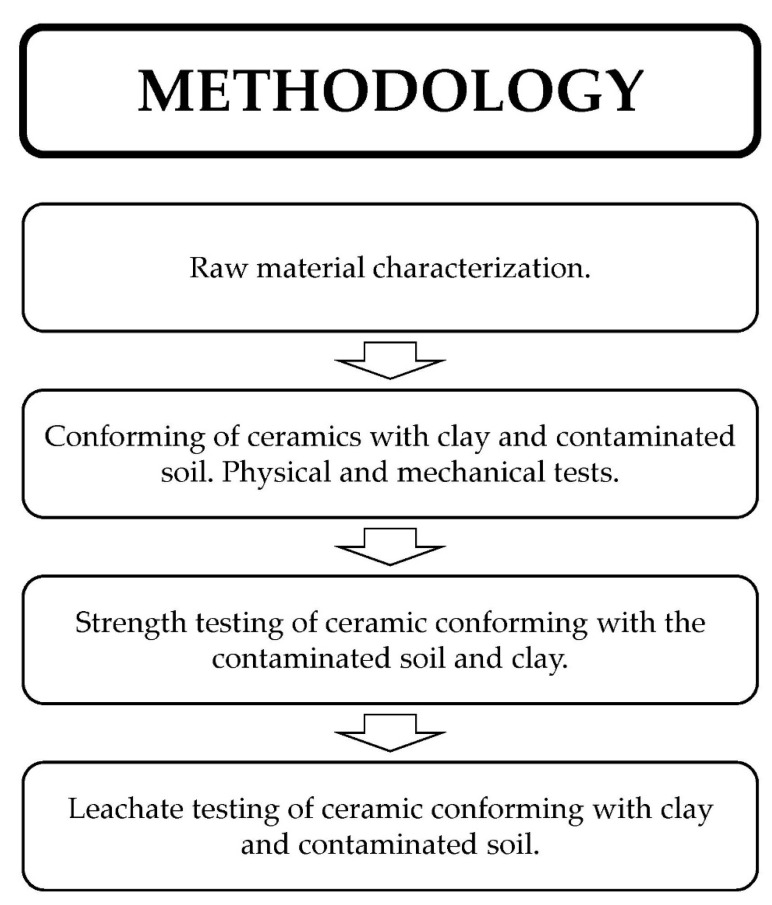
Flow diagram of the different blocks developed in the methodology.

This section describes the tests belonging to the methodology of this research. These tests are divided into four very important blocks for the evaluation of the quality of contaminated soil as a raw material for ceramic materials. These blocks are detailed in Figure 2.

#### 2.2.1. Raw Material Characterization

Initially, in order to determine the compatibility between the clay and the contaminated soil, as well as the presence in the soil of certain elements that could be detrimental to the final manufactured material, a physical and chemical characterization of the clay and the contaminated soil was carried out.

Among the physical tests, the first was the particle density test. In this way, it was possible to evaluate whether the process of mixing and homogenizing the clay with the soil was feasible, since if two materials with very different densities are mixed, it is quite likely that segregation will occur and a homogeneous material will not be obtained. The particle density was evaluated according to the UNE-EN 1097-7 standards [34], determined by the pycnometer method. The liquid limit, plastic limit and plasticity index of the contaminated soil and clay were determined in order to determine the usefulness of the contaminated soil as a material for brick ceramics. The test was carried out by adding different percentages of water to determine the liquid limit and the plastic limit, these values corresponding to the humidity of each test. This is a fundamental property of clay materials that must be evaluated in order to estimate the quality of the conformed ceramic material. This test was carried out according to the UNE-EN ISO 17892-12 standards [35].

Once the fundamental physical properties of the clay and the contaminated soil were determined, the chemical characterization was carried out. The first of the chemical tests carried out was elemental analysis, determining the percentage of carbon, hydrogen and nitrogen in the samples. This test is carried out by analyzing the gases produced in the combustion of the sample at a temperature of 950 ± 5 °C. Therefore, it is capable of identifying and quantifying the aforementioned chemical elements of lower atomic weight. The elemental analysis test was performed with LECO’s TruSpec Micro equipment (TruSpec Micro, LECO, St. Joseph, MI, USA). In turn, the loss on ignition test was carried out at a temperature of 950 ± 5 °C. This test makes it possible to estimate, through the variation in mass that exists in the sample before and after being subjected to the aforementioned temperature, the percentage of carbonates, volatile elements and even the transformation of certain chemical compounds. Finally, in order to identify and quantify the elements with the highest atomic weight in the samples, the X-ray fluorescence test was carried out. This test makes it possible to evaluate the chemical compatibility between the clay and the contaminated soil, the existence of certain harmful elements in the soil for the final conformed ceramic, as well as the existence of potentially toxic elements in the contaminated soil. The determination of these potentially toxic elements in the contaminated soil is essential, since it must subsequently be assessed that the ceramic matrix formed retains the leaching of these elements and, consequently, avoids the possible environmental contamination that this soil may produce.

#### 2.2.2. Conforming of Ceramics with Clay and Contaminated Soil: Physical and Mechanical Tests

Once the contaminated soil and clay were physically and chemically characterized, different ceramic materials were made with both materials. In order to determine the variation of the physical and mechanical properties produced in the ceramic material with the addition of the contaminated soil, a family composed only of clay, a conventional ceramic, was first manufactured. Subsequently, different families of ceramics were made with higher percentages of contaminated soil, in increments of 10%, until the last family composed only with the contaminated soil was obtained. It should be noted that for each family, a total of 6 samples were made, thus obtaining statistically viable results. The different families of ceramics conformed, as well as the percentage of clay and contaminated soil in each one (by mass), are shown in Table 1.

Once the families of ceramic samples to be developed were established, the samples of each of the families were conformed. The process developed was the same for all the families of samples, obtaining comparable results. This process consisted firstly of mixing the contaminated soil and clay in the proportions corresponding to each family until they were homogenized. Subsequently, a percentage of 10% water was added and mixed again until homogenization. The mixture of clay, contaminated soil and water thus prepared was poured into a metal matrix of internal dimensions 60 mm long and 30 mm wide. The mixture poured into the matrix was then applied a pressure of 30 MPa by means of a rammer of similar dimensions. Finally, the material was stripped for its subsequent sintering, drying the different samples for 24 h at a temperature of 105 ± 2 °C. It should be noted that the aforementioned pressure and percentage of water were selected because, as the authors have been able to corroborate, these values allow ceramics with very similar properties to those manufactured industrially to be obtained. 

The sintering process consists of subjecting the different samples to a temperature of 950 ± 5 °C after drying at 105 ± 2 °C for 24 h. To achieve this temperature, a ramp of 4 degrees per minute is used until the temperature of 950 ± 5 °C is reached, and this temperature is maintained for one hour. Finally, the cooling ramp is similar to the ramp up.

The different families of ceramics conformed, as detailed above, were subjected to a series of physical tests to determine the difference produced by the addition of the contaminated soil of a conventional ceramic. The first physical tests carried out were linear shrinkage and weight loss during the sintering process. These tests, carried out according to the UNE-EN 772-16 standards [36], quantify the variation in dimensions and mass that occurs in the material before and after sintering. Therefore, they are essential tests whose results condition other physical parameters such as absorption, porosity and density. In addition, and due to the fact that the final product must have certain dimensions and mass in order to be marketed, these parameters must be taken into account in order to prepare the materials adequately for the industrial process.

Subsequently, the cold water absorption test was carried out in accordance with the UNE-EN 772-21 standards [37]. This test determines the capacity of the conformed ceramic to retain water in its matrix, which is essential for those ceramic elements that are outdoors. In addition, open porosity and bulk density tests were carried out according to the UNE-EN 772-4 standards [38], determining the quality of the structure of the conformed ceramic, as well as the presence of pores that could impair mechanical strength or, on the contrary, benefit thermal insulation and acoustic insulation.

Finally, the color of the different families of ceramics conformed with the clay and the contaminated soil was determined. This property, which is not limited by regulations, is of paramount importance in the marketing of the materials, as the manufacturers want the addition of the waste to vary little in color compared to traditional ceramics. However, certain new colors that may appear as a result of the addition of the waste may be attractive for sale. It is therefore a very subjective physical property that depends mainly on the market. Therefore, in this article only the color coordinates of the different conformed ceramics were measured. This measurement was carried out with the so-called colorimeter, more precisely the commercial device PCE-RGB 2 (RGB-2, PCE, Meschede, Germany).

#### 2.2.3. Strength Testing of Ceramic Conforming with the Contaminated Soil and Clay

Ceramics conformed with clay and contaminated soil are manufactured with the idea of being used in building bricks. Consequently, this element must have an admissible mechanical resistance. The strength of this ceramic element is obviously in simple compression and is regulated according to the UNE-EN 772-1 standards [39]. Therefore, all the families of ceramics were tested according to the aforementioned standards and their simple compressive strength was determined, evaluating which families were feasible to use due to their strength exceeding the 10 MPa set by the European standards. Therefore, this is one of the most restrictive tests, together with the leachate test, to evaluate the maximum percentage of leaching waste incorporation in ceramics.

#### 2.2.4. Leachate Testing of Ceramic Conforming with Clay and Contaminated Soil

Finally, in order to evaluate the retention of potentially toxic elements in the ceramic matrix and, consequently, the toxicity of the different families of ceramics and their potential to leach contaminants, the TCLP test [1] was performed on all families of ceramics. For this purpose, the ceramics were ground to obtain samples with a particle size of less than 10 mm. According to the EPA standards [1], the fluid composed of glacial acetic acid (5.7 mL) and 64.3 mL of sodium hydroxide solution (1 N) diluted in 1 L of distilled water, was 20 times the mass of the sample. The mixture of the sample and the prepared solution was stirred for 18 ± 2 h at a temperature of 22 ± 3 °C. The solution was then filtered through a glass fiber filter with an effective pore size of 0.7 micrometers and acidified with nitric acid to a pH of 2. The extracted liquid was analyzed by inductively coupled plasma mass spectrometry (7900, Agilent, Santa Clara, CA, USA).

In this way, the leaching of potentially toxic elements in the different families of ceramics could be estimated, establishing a limit of incorporation of the contaminated soil for ceramic conforming bricks.

## 3. Results and Discussion

This section describes the results of the tests mentioned in the methodology, as well as the partial discussions of these results that were obtained.

### 3.1. Raw Material Characterisation

First, the physical and chemical characterization of the contaminated soil and clay was carried out. The first of the tests carried out for the physical characterization was the particle density test, with the values shown in Table 2 below.

The particle density of contaminated soil and clay is very similar. Therefore, there should not be any problems in the mixing process of both materials that would lead to the materials segregation and inhomogeneous mixing. In order to determine the quality of the materials analyzed for use in ceramics, the liquid limit, plastic limit and plasticity index of the soil and clay were calculated. These results are shown in Table 3.

As can be seen in Table 3, the plasticity index of clay is ideal for ceramic conforming materials. This fact is reflected in the number of ceramic industries using this material. In turn, the plasticity index of contaminated soil is much lower than that of clay, reflecting the fact that ceramics conformed with this material at the set sintering temperature will develop a lower quality ceramic material. Consequently, the addition of the contaminated soil in the ceramic according to its plasticity index will create a ceramic with substantial variations with respect to the one made only with clay. 

On the other hand, for the chemical characterization, the elemental analysis test was carried out first, determining the percentage of carbon, nitrogen and hydrogen in the contaminated soil and clay. The results of the elemental analysis test are shown in Table 4.

The contaminated soil shows elemental analysis test results very similar to those obtained for the clay. The percentage of nitrogen in both materials is low, and consequently there are no chemical compounds with this element that could cause environmental contamination. At the same time, the percentage of carbon and hydrogen in both materials is equally low, showing that the percentage of organic matter in both samples is also low. The carbon identified may belong mainly to the carbonate compounds of the materials and the hydrogen to the hydrated compounds.

The loss on ignition test, in turn, determined the mass variation that occurred before and after subjecting the contaminated soil and clay to a temperature of 950 ± 5 °C. The results of this test are shown in Table 5.

The loss on ignition of the contaminated soil is relatively low, even lower than that of the clay. This mass variation confirms that in both materials the percentage of organic matter is low (as determined in the elemental analysis test), and the mass variation in both materials may correspond to the loss of volatile elements other than carbon, nitrogen and hydrogen, as well as to the transformation of certain chemical compounds. In all cases, the loss on ignition values of both materials are suitable for use in ceramic materials.

Finally, in order to identify and quantify all chemical elements of higher atomic weight existing in the contaminated soil and clay, the X-ray fluorescence test was performed. The results of this X-ray fluorescence test are shown in Table 6.

The X-ray fluorescence test shows compatibility between the contaminated soil and the clay, as both elements appear to be silicate-aluminates. This is reflected in the percentage of silicon and aluminum in both materials, as well as the low percentage of carbonates determined by the elemental analysis test, so much so that the percentage of iron in the contaminated soil and the clay are practically the same. However, the higher percentage of calcium present in the soil will create a ceramic of lower quality than that produced with the clay. It is worth mentioning the existence in the contaminated soil of potentially toxic elements such as zinc, lead, barium and arsenic. These chemical elements can cause significant environmental pollution if they are not treated and, obviously, they are only present in the contaminated soil and not in the clay. It is mainly these chemical elements, together with cadmium, which must be retained in the ceramic matrix, and which were evaluated in this research to verify that their immobilization in the final material takes place.

### 3.2. Conforming of Ceramics with Clay and Contaminated Soil: Physical and Mechanical Tests

Once the contaminated soil and clay were physically and chemically characterized, the different families of ceramics were conformed with both materials. These families of ceramics are detailed in Table 1. The manufacturing procedure was carried out according to the specifications detailed in the methodology. The different ceramics conformed were subjected to the aforementioned physical tests, obtaining the results detailed below.

**Figure 3:**
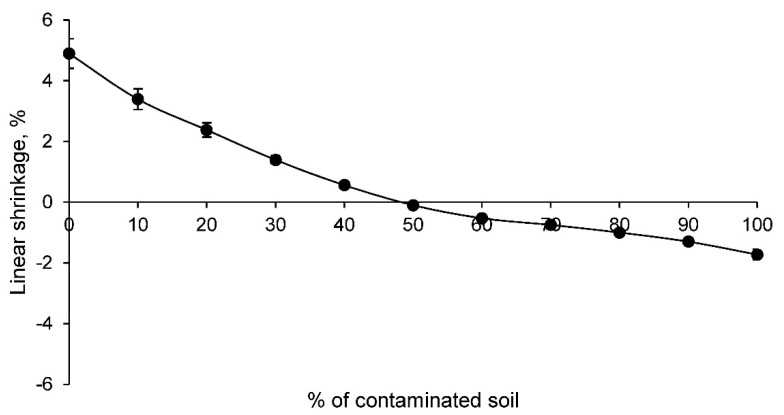
Linear shrinkage of families of ceramics conforming with various percentages of clay and contaminated soil.

The first of the physical tests performed was the linear shrinkage test. The results of this test for all the families of ceramics conformed with the contaminated soil and clay are shown in Figure 3.

The linear shrinkage of the ceramics decreases as the percentage of contaminated soil increases. Consequently, after the incorporation of 50% contaminated soil, a linear expansion occurs during the sintering process of the ceramic. This linear expansion is due to several factors, including the particle size of the contaminated soil. A larger particle size in the contaminated soil will prevent the formation of a denser ceramic, as the bonding of these particles in the sintering process is not as excellent as it is with clay. Therefore, this behavior reflects that the structure of the ceramics with contaminated soil, which is produced in the sintering process, is a more open structure. Furthermore, it will condition the following physical and mechanical properties of all families.

**Figure 4:**
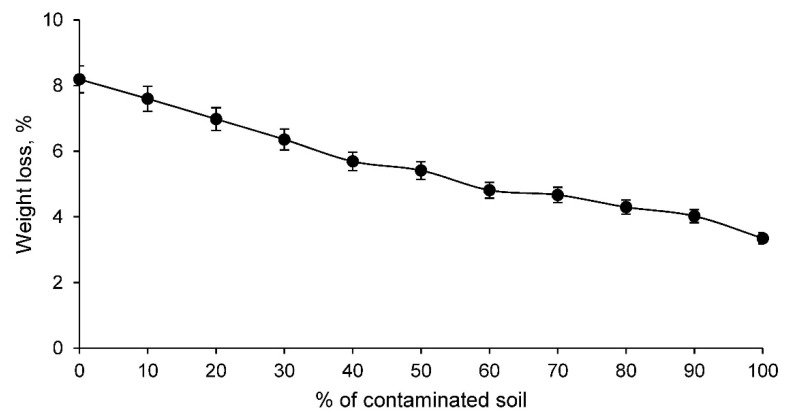
Weight loss of conforming ceramics families with various percentages of clay and contaminated soil.

At the same time, the weight loss test of the different families of ceramics during the sintering process was carried out. The results of this test are shown in Figure 4.

Figure 4 shows that the weight loss of the conformed ceramics is lower the higher the percentage of contaminated soil. This is to be expected if one takes into account the loss on ignition of the contaminated soil and the clay, whose values coincide with the family of ceramics conformed only with the contaminated soil and the clay. Consequently, the weight loss of the intermediate ceramic families with varying percentages of clay and soil are practically proportional to the loss on ignition of both materials. 

**Figure 5:**
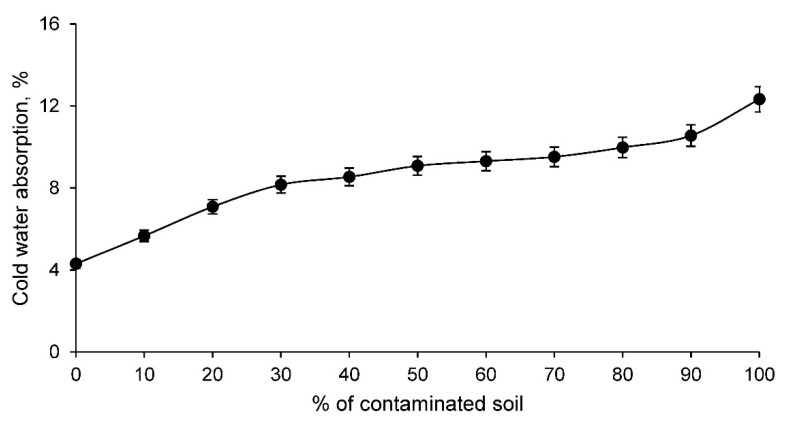
Cold water absorption of conforming ceramics families with various percentages of clay and contaminated soil.

On the other hand, the cold water absorption of the ceramic conforming families is shown in Figure 5.

**Figure 6:**
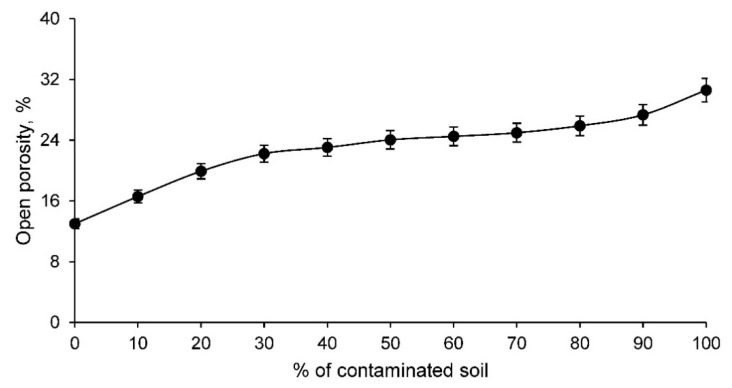
Open porosity of the families of ceramics conforming with various percentages of clay and contaminated soil.

The cold water absorption of the different families of clay and contaminated soil conformed ceramics increases as the percentage of contaminated soil increases. This is to be expected if the linear shrinkage results are taken into account. Therefore, this test shows that ceramics with contaminated soil present a more open structure capable of absorbing a higher percentage of water under normal conditions. This fact is very interesting and should be taken into account for ceramic elements that are exposed to the weather, since these materials can absorb rainwater and overload the weight of the structure that supports them. However, this higher water absorption also reflects the fact that there is obviously a higher porosity, conditioning other very interesting characteristics for the construction elements. To corroborate this increase of pores in the ceramic, the open porosity test was carried out for all the families of ceramics conformed with clay and contaminated soil, reflecting the results shown in Figure 6.

The open porosity of the ceramic increases as the percentage of contaminated soil increases. This fact implies that there is a more open structure in ceramics with contaminated soil that conditions a lower mechanical strength. However, this greater porosity, as long as the specifications of the regulations are met, can condition interesting characteristics of the material such as acoustic insulation and thermal insulation. Both characteristics, all other things being equal, increase the greater the porosity of the ceramic.

**Figure 7:**
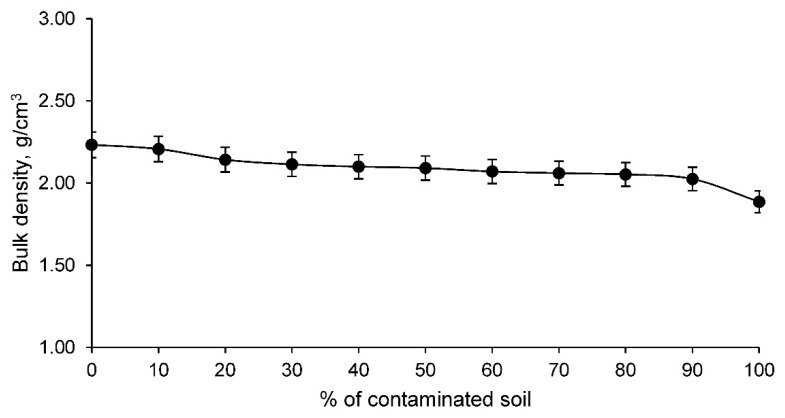
Bulk density of the families of ceramics conforming with various percentages of clay and contaminated soil.

In addition, an essential property for any material was determined: the bulk density. The bulk density of the ceramics conformed with clay and contaminated soil are shown in Figure 7.

The bulk density of ceramics conformed with clay and contaminated soil, as shown in Figure 7, decreases as the percentage of contaminated soil increases. It should be noted that this bulk density is not similar to the particle density, the latter being larger the higher the percentage of contaminated soil, as this element has a higher particle density. Therefore, the bulk density reflects the fact that the ceramics have a more open structure with a greater number of pores. This magnitude conditions mechanical aspects and other physical properties such as insulation, as detailed above.

**Figure 8:**
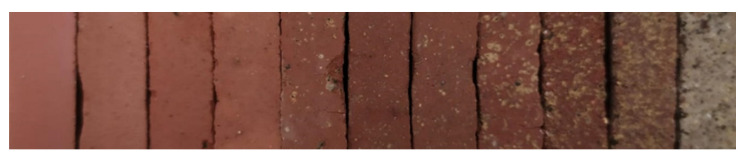
Image of the ceramics conformed with various percentages of clay and contaminated soil. From left to right, CS0, CS1, CS2, CS3, CS4, CS5, CS6, CS7, CS8, CS9 and CS10.

Finally, and with the sole objective of recording the color in a scientific and rigorous manner, the colorimetric test was carried out. This physical property, as mentioned above, is very subjective. However, it is true that most companies that market ceramics do not want the incorporation of the waste to considerably change the color of the ceramic. Therefore, it is a property that should at least be quantified, although it is the market that will later decide whether the color is appropriate or not. Figure 8 shows an example of each family of conformed ceramics, from the left showing the family made only with clay (CS0) to the right showing the family conformed with contaminated soil and without clay (CS10).

As mentioned in the methodology, the color coordinates for rigorous recording were measured with a colorimeter. The color coordinates of all families of ceramics conformed with clay and the contaminated soil are shown in Table 7.

Consequently, the physical tests carried out on the various families of ceramics with the contaminated soil and clay show that in all cases acceptable results are obtained in accordance with the regulations. Therefore, it is not these tests that limit the maximum percentage of contaminated soil to be incorporated. Furthermore, the combination of contaminated soil and clay makes it possible to obtain ceramics with particular properties of porosity, color and water absorption that are very interesting for specific applications. Therefore, there are a number of materials with different properties created only with the combination of two raw materials.

### 3.3. Strength Testing of Ceramic Conforming with the Contaminated Soil and Clay

**Figure 9:**
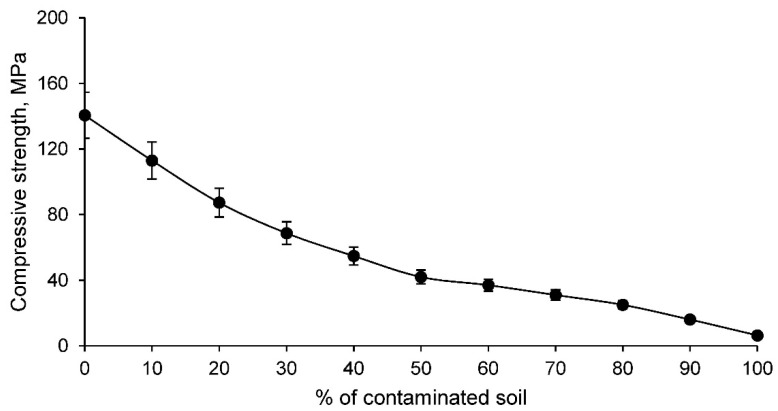
Simple compressive strength of families of ceramics conformed with various percentages of clay and contaminated soil.

The manufactured ceramics are intended for use in building bricks. Consequently, these ceramics must have an admissible simple compressive strength according to the standards. In order to determine this property of the ceramics, the simple compressive strength test was carried out for all families, with the results shown in Figure 9.

The simple compressive strength of the conformed ceramics decreases as the percentage of contaminated soil increases, which is to be expected if it is taken into account that the linear shrinkage and density of the ceramics decreases as the percentage of contaminated soil increases. According to the limitations set by the building brick standards, which states that the strength must be higher than 10 MPa, it can be stated that ceramics conformed with clay and percentages of contaminated soil percentages of 90% and 100% are unacceptable. Therefore, the CS9 and CS10 families detailed in Table 1 are not suitable for use in ceramic materials intended for bricks.

### 3.4. Leachate Testing of Ceramic Conforming with Clay and Contaminated Soil

The aim of this research is to prevent contaminated soil, mainly with heavy metals, from causing environmental pollution. Therefore, this contaminated soil has been added in different percentages with clay to conform different families of ceramics. These ceramics have been evaluated physically and mechanically, determining their suitability for use. However, it is essential to corroborate that the pollutants in the contaminated soil do not produce a leachate with high concentrations of these elements. For this purpose, the TCLP test [1] was developed for all families of ceramics. The leachates thus produced must comply with the limitations established by the EPA [1] for the chemical elements under study. These limits are shown in Table 8.

**Figure 10:**
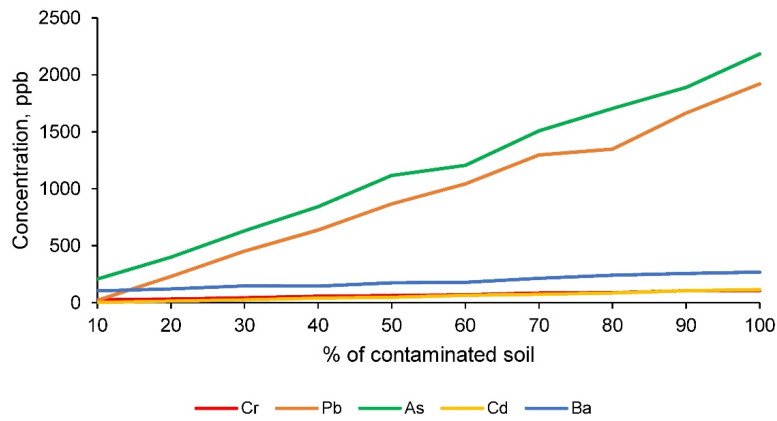
Concentration of toxic elements in the leachate, obtained according to the TCLP leaching test, for the families of clay and contaminated soil conformed ceramics.

The TCLP test [1] results, performed on all families of conformed ceramics, reflect the leachate concentrations shown in Figure 10 for the chemical elements assessed by the EPA [1].

The results obtained from the leaching test according to the TLCP methodology [1], for the chemical elements chromium, lead, arsenic, cadmium and barium, show that all the families of ceramics conformed comply with the permissible limits established by the EPA [1]. Consequently, it can be affirmed that this test is not limiting for the maximum incorporation of contaminated soil and that the ceramic material has encapsulated the toxic elements under study in its matrix, as desired.

**Figure 11:**
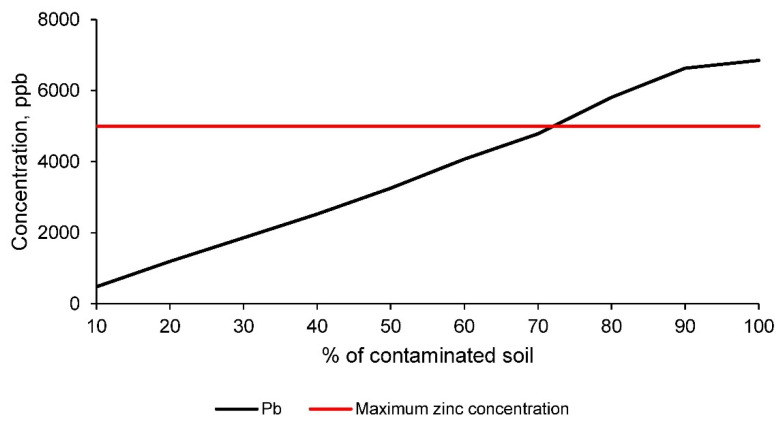
Zinc concentration in the leachate, obtained according to the TCLP leaching test [1], for the different families of ceramics, conformed with clay and the contaminated soil, compared to the maximum permissible limit established by the EPA for domestic water [40].

However, it should be taken into account that there is no information for the chemical element zinc and that this element represents an important percentage in the contaminated soil. Therefore, in order to establish an even stricter comparison, a more restrictive standard for this chemical element, zinc, was applied. The standard used was that for drinking water for human consumption, which sets the maximum concentration of zinc at 5000 ppb. This standard, as mentioned above, is much stricter and does not at all coincide with the purpose of the ceramics. Nevertheless, this regulation has been applied to verify the retention of this chemical element in the matrix. Therefore, the results of zinc concentration in the leachate according to the TCLP method [1], as well as the maximum allowed by the mentioned regulation, are shown in Figure 11.

Figure 11 shows how the concentration of zinc in the leachate of the different families of ceramics conformed with clay and the contaminated soil is lower, up to the incorporation of 70% of soil, than the limit set by the regulations for drinking water for human consumption. Although these regulations are much more restrictive than necessary to corroborate the suitability of the ceramics, in this research it has been established that the maximum percentage of incorporation of contaminated soil for the manufacture of ceramics is 70%. Therefore, all families with lower percentages of contaminated soil obtain acceptable physical, mechanical and contaminant elements retention properties.

## 4. Conclusions

The tests mentioned in the methodology allow us to obtain a series of partial conclusions that lead to the verification of the final objective of this research. This objective is to reuse soil contaminated by mining activities for the shaping of ceramic materials that avoid, in turn, the contamination that could be caused by leaching of potentially toxic elements existing in the soil. For this reason, the following is a summary of the partial conclusions obtained in this research.

- The density of the contaminated soil is very similar to the density of the red clay used. However, the soil has a lower plasticity index. On the chemical side, the nature of both samples is very similar, both being silicate-aluminates with a low percentage of carbonates and organic matter. However, the contaminated soil has a series of contaminating elements such as zinc, lead, barium and arsenic that must be retained in the ceramic matrix.
- Ceramics conformed with contaminated soil and clay show lower linear shrinkage and bulk density as the percentage of contaminated soil increases in the material. At the same time, an increase in cold water absorption and porosity is observed with the higher percentage of contaminated soil. Consequently, it can be stated that the addition of contaminated soil creates a ceramic material with a more open structure than a traditional ceramic.
- The mechanical strength of clay and contaminated soil conformed ceramics decreases as the percentage of contaminated soil increases, obtaining unacceptable simple compressive strength values for contaminated soil percentages higher than 80%.
- The TCLP test [1] carried out to assess the immobilization of potentially toxic elements showed, according to EPA regulations [1], that all ceramic families were suitable for use. However, when applying the stricter drinking water standards for human consumption, it was found that the families of ceramics conformed with soil contamination percentages above 70% were unacceptable by the chemical element zinc.

Consequently, and according to the partial conclusions obtained, it can be stated that it is possible to use contaminated soil from the mining district of Linares, Spain, as a raw material for ceramic materials up to a percentage of 70%. In this way, the contamination that can be caused by soil contaminated by mining activities is eliminated, this soil is given a new useful life, the extraction of new raw materials is avoided, and the contamination produced by the leaching of the contaminating elements present in the soil is cancelled. This research is therefore an example of the potential that still exists in mining waste and of the different industrial activities that can be developed to avoid the environmental impact of the mining industry.

## Figures and Tables

**Table 1:** Families of conformed ceramics with different combination percentages of clay and contaminated soil.

Family	Clay, %	Contaminated Soil, %
CS0	100	0
CS1	90	10
CS2	80	20
CS3	70	30
CS4	60	40
CS5	50	50
CS6	40	60
CS7	30	70
CS8	20	80
CS9	10	90
CS10	0	100

**Table 2:** Particle density of the contaminated soil and clay.

Sample	Density, g/cm^3^
Contaminated soil	2.790 ± 0.066
Clay	2.443 ± 0.063

**Table 3:** Liquid limit, plastic limit and plasticity index of the contaminated soil and clay.

	Contaminated Soil	Clay
Liquid limit	18.3 ± 0.4	38.5 ± 1.4
Plastic limit	11.2 ± 0.3	22.1 ± 0.6
Plasticity index	7.1 ± 0.1	16.4 ± 0.6

**Table 4:** Elemental analysis test of the contaminated soil and clay.

Sample	Nitrogen, %	Carbon, %	Hydrogen, %
Contaminated soil	0.171 ± 0.005	1.614 ± 0.062	0.226 ± 0.008
Clay	0.040 ± 0.001	1.164 ± 0.040	0.651 ± 0.023

**Table 5:** Loss on ignition of the contaminated soil and clay.

Sample	Loss on Ignition, %
Contaminated soil	2.94
Clay	7.90

**Table 6:** X-ray fluorescence of the contaminated soil and clay.

Element	Contaminated Soil. Wt, %	Clay. Wt, %
Si	31.24 ± 0.11	24.60 ± 0.12
Fe	5.77 ± 0.1	5.48 ± 0.09
Ca	4.61 ± 0.09	2.28 ± 0.06
Al	3.04 ± 0.06	9.44 ± 0.10
K	2.04 ± 0.06	4.67 ± 0.10
Na	1.31 ± 0.05	0.123 ± 0.011
Zn	1.08 ± 0.05	-
Sx	0.446 ± 0.02	0.0247 ± 0.0013
Mg	0.543 ± 0.027	2.07 ± 0.05
Pb	0.649 ± 0.032	-
Ba	0.491 ± 0.05	-
Ti	0.148 ± 0.0074	0.461 ± 0.023
As	0.142 ± 0.021	-
Mn	0.136 ± 0.0068	0.119 ± 0.0059
Px	0.0459 ± 0.0025	0.0672 ± 0.0034
Cu	0.061 ± 0.003	-
Sr	0.0398 ± 0.0028	0.0291 ± 0.0030
Zr	0.031 ± 0.0035	0.0280 ± 0.0036
Cl	0.0266 ± 0.0013	0.0095 ± 0.0008
Cr	0.0133 ± 0.0015	0.0112 ± 0.0016
Ni	0.0147 ± 0.0016	0.0183 ± 0.0016
V	-	0.0200 ± 0.0017
Ru	-	0.0242 ± 0.0016
Rb	-	0.0250 ± 0.0044
Pd	-	0.0237 ± 0.0035
Pt	-	0.0158 ± 0.0034
Co	-	0.0057 ± 0.0017

**Table 7:** Color coordinates (Red, Green and Blue) of the different families of ceramics.

Family	Red	Green	Blue
CS0	280	163	125
CS1	275	162	125
CS2	274	167	131
CS3	261	157	123
CS4	253	155	121
CS5	276	169	127
CS6	256	161	120
CS7	239	152	116
CS8	238	158	118
CS9	245	190	145
CS10	361	303	257

**Table 8:** Maximum concentrations of metals or toxic elements in the leachate according to the TCLP method [1].

Metals	Maximum Allowable Concentration in the Leachate, ppb
Cr	5000
Pb	5000
As	5000
Cd	1000
Ba	100,000

## Data Availability

Data is contained within the article.
